# Antioxidant, Antifungal Activities of Ethnobotanical *Ficus hirta* Vahl. and Analysis of Main Constituents by HPLC-MS

**DOI:** 10.3390/biomedicines8010015

**Published:** 2020-01-15

**Authors:** Chuying Chen, Xuan Peng, Jinyin Chen, Chunpeng Wan

**Affiliations:** 1 Jiangxi Key Laboratory for Postharvest Technology and Nondestructive Testing of Fruits and Vegetables, Collaborative Innovation Center of Postharvest Key Technology and Quality Safety of Fruits & Vegetables in Jiangxi Province, College of Agronomy, Jiangxi Agricultural University, Nanchang 330045, China; cy.chen@jxau.edu.cn; 2 Pingxiang University, Pingxiang 337055, China; pengx1104@163.com

**Keywords:** *Ficus hirta* Vahl, antioxidant, antifungal activity, agar dilution, agricultural products

## Abstract

The medicinal and edible plant, *Ficus hirta* Vahl. (also called hairy fig), is used for the treatment of constipation, inflammation, postpartum hypogalactia, tumors, and cancer. There is an urgent need for scientific evaluation to verify the pharmacological properties of *F. hirta*. Therefore, in vitro assays evaluated the antioxidant and antifungal activities of various solvent extracts of hairy fig fruits (HFF). HFF extracts had abundant antioxidant components for a significant amount of total phenolic (TPC) and flavonoid contents (TFC) (TPC from 17.75 ± 0.52 to 85.25 ± 1.72 mg gallic acid/g dw and TFC from 15.80 ± 0.59 to 144.22 ± 8.46 mg rutin/g dw, respectively). The ethyl acetate extract (EAE) and acetone extract (AE) of HFF demonstrated potent antioxidant activities against 1,1-diphenyl-2-picrylhydrazyl (DPPH) (IC_50_ values of 2.52 and 2.02 mg/mL, respectively) and ABTS radicals (IC_50_ values of 3.06 and 9.26 mg/mL, respectively). Moreover, the AE with a high TFC showed a prominent in vitro and in vivo antifungal activity against *Penicillium italicum*, causing citrus blue mold. Eighteen metabolites were identified or putatively identified from six HFF extracts. Current findings indicated that HFF extracts had significant antioxidant and antifungal activities and could potentially be used as an alternative agent for the preservation of agricultural products.

## 1. Introduction

Hairy fig fruits (*Ficus hirta* Vahl. Family Moraceae) can easily be identified due to their five-fingered leaves. Mature fruits resemble wild peaches and have been utilized as medicine and food for centuries [1,2]. They are widely distributed and commercially grown in southern China for meeting the food demands along with the treatment of constipation, inflammation, postpartum hypogalactia, tumors, and cancers [3,4,5,6]. There are hardly any scientific studies about the antioxidant and antifungal activities of hairy fig fruits (HFF). The HFF is a famous herbal medicine known in China by the name of ‘Wú Zhǐ Máo Táo Guǒ’, and has been used in both medicine and food for centuries by Hakka people [1,7,8].

The in vitro antimicrobial properties of roots and fruits from *F. hirta* Vahl. against *Escherichia coli*, *Staphylococcus aureus*, *Alternaria citri*, *Botrytis cinerea*, and other pathogens have been extensively reported [9,10]. Moreover, the crude extracts (aqueous, ethyl acetate, and butyl alcohol) of HFF exhibit cytotoxic effects on HeLa cells [6]. Certain studies regarding the chemical composition and pharmacological activities of *F. hirta* Vahl. reported that benzene derivatives, phenolics, and glycosides of flavonoid are majorly present in this plant [2,5,11,12]. Recently, three new monosubstituted benzene derivatives elucidated as (*2R*) methyl 2-*O*-*β*-d-glucopyranosyl-2-phenyl acetate, (2*S*) 2-*O*-benzoyl-butanedioic acid-4-methyl ester, and 4-*O*-benzoyl-quinic acid were initially isolated from the ethanolic extracts of HFF by our group members [8], and one new compound depicted a potent antifungal effect against *Penicillium italicum* isolated from citrus fruits. Continuing this research, our group members further isolated another three glycosides of flavonone, and pinocembrin-7-O-β-d-glucoside (PCBG) was said to be a major flavonoid in HFF and showed a prominent in vitro antifungal activity against *P. italicum* and *P. digitatum* [11,13]. The antifungal mechanism revealed by metabolomics showed that amino acids, lipids, tricarboxylic acid cycle, and ribonucleic acids all participated in the antifungal process [13]. Furthermore, 5-O-[*β*-d -apiofuranosyl-(1→2)-*β*-d-glucopyranosyl]-bergaptol was the first ever reported furanocoumarin glycoside extracted from the roots of *F. hirta* Vahl. [12]. Several studies also claimed an anti-inflammatory activity of the root extracts of *F. hirta* Vahl., and phenylpropanoids, bergapten, lupeol palmitate, and azelaic acid were responsible for the anti-inflammatory activity [4,14,15]. However, as far as current literature survey is concerned, there are no scientific studies describing the amount of total phenolic and flavonoid contents correlated with antioxidant and antifungal activities. The antioxidant and antifungal properties of *F. hirta* Vahl. fruit extracts obtained by various solvents have not yet been reported. Therefore, the present study was designed to examine the effects of various solvent extracts on the total phenolic and flavonoid contents along with antioxidant and antifungal activities of HFF using in vitro model systems, and the chemical constituents were also analyzed by high-performance liquid chromatography−mass spectrometry (HPLC-MS).

## 2. Materials and Methods

### 2.1. Collection of Plant Materials and Reagents

Hairy fig fruits (HFF) were purchased from the Huafeng herbal store in Zhangshu City (Jiangxi Province, China). The HFF samples were ground into powder by using an electric grinder, dried below 45 °C for 15 h, sieved by using number 20 mesh, and finally stored in a hermetically sealed bag at 4 °C for later use.

Folin–Ciocalteu reagent and 1,1-diphenyl-2-picrylhydrazyl (DPPH) were bought from Solarbio (Beijing, China). Gallic acid, rutin, and ascorbic acid were bought from the Institute of Biological Products (Beijing, China). Chloroform, petroleum ether, acetone, methanol, and ethyl acetate used in the current study were purchased from Sinopharm Chemical Reagents Co., Ltd. (Beijing, China). All chemicals and reagents used in the present study were purely of analytical grade.

The fifteen standard materials of methyl-1,2,3,4-tetrahydro-*β*-carboline-3-carboxylic acid, methyl-1-methyl-1,2,3,4-tetrahydro-*β*-carboline-3-carboxylate, dihydrophaseic acid, vomifoliol, dehydrovomifoliol, pubinernoid A, 2-phenylethyl-*O-β*-d-glucoside, 1-*O*-trans-cinnamoyl-*β*-d-glucopyranosyl-(1→6)-*β*-d-glucopyranoside, 4-O-benzoyl-quinic acid, 3-*O*-benzoyl-quinic acid, benzyl-β-d-glucopyranoside, (2S) 2-*O*-benzoyl-butanedioic acid-4-methyl ester, pinocembrin-7-*O*-*β*-d-glucoside, naringenin-7-*O*-*β*-d-glucoside, and eriodictyol-7-*O*-*β*-d-glucoside were isolated and identified by nuclear magnetic resonance (NMR) and high resolution electrospray ionization mass spectrometry (HR-ESI-MS), as previously described [8,11].

### 2.2. HFF Extracts Preparation

The dried powder of HFF (30 g) was extracted using 1 L of different solvents viz., chloroform (CE), petroleum ether (PEE), acetone (AE), methanol (ME), distilled water (WE), and ethyl acetate (EAE), under an ultrasonic wave at 40 kHz for 60 min at 25 °C. All hairy fig fruits extracts (HFFE) were filtered using filter paper (30–50 μm pore size range) in a Buchner funnel, while the residual extracts were filtered two times using corresponding solvents (500 mL), as described above. All organic filtrates were removed using a rotary evaporator (Buchi Rotavapor R-3, Flawil, Switzerland) to obtain dry extract. The filtrates of the water extract were rapidly put in a freezer (−40 °C) and let to dry using a vacuum freeze dryer for 48 h. The accumulative yield of the HFF extracts is tabulated in Table 1. All extracts were dried and put into −20 °C and reconstituted with the corresponding solvents or distilled water to obtain the desired concentration for further analyses.

### 2.3. Estimation of Total Phenolic Contents

The total phenolic contents (TPC) in various HFF (PEE, CE, EAE, AE, ME, and WE) extracts were measured following a slightly modified Folin–Ciocalteu method given by Wan and colleagues [16]. Briefly, the substrates were mixed with 0.1 mL of each extract solution and 5 mL of 10-fold diluted Folin–Ciocalteu phenol reagent. Three minutes later, 1.5 mL of 20% (*w*/*v*) sodium carbonate and 8 mL of distilled H_2_O were added into the mixture. This mixture was kept at 25 °C for 2 h, and an absorbance measurement was taken at 765 nm with a microplate reader (M5 Multiscan Spectrum, Molecular Devices Corporation, Sunnyvale, CA, USA). The TPC was calculated by using the gallic acid calibration curve and represented as mg GAE/g dry HFF extract. The calibration equation for GA was Y = 14.218X + 0.022 (*R*^2^ = 0.9995).

### 2.4. Estimation of Total Flavonoid Contents

The total flavonoid contents (TFC) in various HFF extracts were analyzed using the aluminum chloride method [17] with few modifications. Briefly, 1.0 mL of each extract solution was mixed with 2.0 mL of 0.1 mM aluminum chloride, 1.0 mL of 1 M sodium acetate (pH 5.2), and 6.0 mL of methanol. These mixtures were placed in a water bath at 40 °C for incubation of about 10 min, and the absorbance measurements were taken at 421 nm using a microplate reader (M5 Multiscan Spectrum, Molecular Devices Corporation, Sunnyvale, CA, USA). Rutin (0–80 mg/L in methanol) was used as a standard, and the TFC was represented as mg rutin equivalent (RE)/g dry HFFE. The following calibration equation was used for rutin. Y = 0.1192X – 0.1225 (*R*^2^ = 0.9991).

### 2.5. In Vitro Evaluation of Antioxidant Activity

#### 2.5.1. DPPH assay

The activities in various HFF extracts were carried out following a previous method [18] with few alterations. Initially, 2.9 mL of 0.06 mM DPPH dissolved in ethanol was mixed with 100 μL of each extract (concentrations ranged from 2 to 10 mg/mL). The absorbance of the mixtures was determined at 517 nm after 30 min of reaction incubation in the dark at 25 °C with a M5 Multiscan Spectrum microplate reader (Molecular Devices Corporation, Sunnyvale, CA, USA). The DPPH free radical-scavenging activity was measured and expressed as a percentage using the equation described previously [18]. All experimental procedures were repeated as three independent experiments. Ascorbic acid (AsA, 0.02, 0.04, 0.06, 0.08, 0.10, 0.15, and 0.20 mg/mL) was used as a positive control. The DPPH radical-scavenging activity was calculated and expressed as a percentage using the following equation:

$$\mathrm{Scavenging}\;\mathrm{effect}\;(\%)\;=\;\frac{A_{0}-A_{t}}{A_{0}}\times 100$$

where A_0_ and A_t_ are the absorbance of the control (without extract) and HFF extract added after 30 min of reaction incubation, respectively.

#### 2.5.2. ABTS Assay

The assay of HFF extracts to decolorize 2,2′-azino-bis-(3-ethylbenzothiazoline-6-sulphonic acid (ABTS) radicals was followed, with few modifications [19]. Briefly, the ABTS solution was formed by adding 7 mM ABTS solution mixed with 2.45 mM potassium persulfate and left to complete the reaction in the dark at 25 °C for 15 h. A total of 2.7 mL of the ABTS solution was mixed with 300 μL of each extract (concentrations ranged from 2 to 10 mg/mL). The measurement of the absorbance of the resultant mixture was done at 734 nm after 6 min of reaction in the dark at 25 °C. All samples were run in triplicate. The same procedure was done for ascorbic acid (concentrations ranged from 0.5 to 2.5 mg/mL) as a positive control. The ABTS radical-scavenging activity was formulated and expressed as a percentage using the above equation.

Here, A_0_ and A_t_ are the absorbance of the ABTS solution and HFF extract added after 6 min of reaction incubation, respectively.

#### 2.5.3. Ferric Reducing Antioxidant Potential (FRAP) Assay

The FRAP assay of HFF extracts was applied with some modifications to reduce ferric ions (Fe^3+^) [17]. Briefly, the mixture containing 1.0 mL of 50 mM phosphate buffer (pH 6.6) and 1.0 mL of 1% (*w*/*v*) potassium ferricyanide was added to 0.5 mL of each extract (concentrations ranged from 2 to 10 mg/mL). A total of 1.0 mL of 10% (*w*/*v*) trichloroacetic acid (TCA) was added to the mixture after incubating at 50 °C for 20 min, and spun at 3000 rpm for 10 min. A total of 1.5 mL of the supernatant was thoroughly mixed with 1.5 mL distilled H_2_O and 0.3 mL of 0.1% (*w*/*v*) ferric chloride in a test tube. The absorbance of the mixture was determined at 700 nm using distilled H_2_O as the blank solution after keeping it at 25 °C for 10 min. The same was done for AsA as a positive control. All experimental procedures were repeated as three independent experiments.

### 2.6. Antifungal Activity of HFF Extracts

#### 2.6.1. Fungi Strains and Growth Condition

To examine the antifungal potential of HFF extracts, a total of six different fungal strains including *Penicillium italicum* (CGMCC 3.4040), *P. digitatum* (CGMCC 3.15410), *Aspergillus niger* (CGMCC 3.17612), *A. oryzae* (CGMCC 3.13905), *Saccharomyces cerevisiae* (CGMCC 2.3866), and *Candida utilis* (CGMCC 2.2917) were tested in antifungal assays. These fungal strains were bought from China General Microbiological Culture Collection Center (Beijing, China).

The stock cultures were maintained on plate count agar at 4 °C. Fungal strains were cultured for 48 h at 25 °C in potato dextrose agar (PDA, 200 g of boiled potato extract, 20 g of glucose, 20 g of agar powder, and 1000 mL distilled H_2_O). All tested pathogenic strains were standardized to a concentration of 10^7^ cfu/mL for antifungal activity test.

#### 2.6.2. In vitro Antifungal Assay

The modified method of Bauer–Kirby disk tests was used for measuring zones of the antimicrobial activities of HFF extracts [20]. Petri dishes (diameter, 90 mm) were prepared with PDA medium (about 15 mL) and surface inoculated with the optimal concentration of spore suspensions in sterile water. A sterile Oxford cup (diameter, 8 mm) was impregnated with 200 μL of each extract. The diameters of inhibitory zones around the Oxford cups were measured in mm after 48 h of culture at 25 °C for fungal strains under darkness. The extract was considered to be a potential antimicrobial agent when the diameter of the inhibitory zone was larger than 8 mm. Natamycin (at the concentration of 0.05 mg/mL) was used as the standard fungistat. All experiments were evaluated in quadruplicate in this assay.

The MICs of six HFF extracts on the mycelial growth of the tested fungal strains were determined using the agar dilution method described previously [21]. Different concentrations of HFF extracts were mixed with PDA in a proportion of 1:9 for obtaining the final concentrations of 0, 62.5, 125, 250, 500, 1000, and 2000 µg/mL. The 6 mm diameter mycelial disks cut from a 7-day-old culture of the tested fungal strains were placed in the center of each Petri dish, and incubated at 25 °C. The MIC was defined as the lowest HFF extracts’ concentration that 100% inhibited the growth of the tested fungal strains after 48 h of culture.

#### 2.6.3. In Vivo Antifungal Assay on Mycelial Growth of *P. Italicum*

Navel orange (*Citrus Sinensis* L. Osbeck cv. Lane Late) fruits were harvested at commercial maturity (soluble solids content of 12.0–12.5%) from Sanbaishan orchard, situated in Ganzhou City, China. The healthy fruits having a consistent size (240–280 g), uniform color (citrus color index, 4.8–6.0), and free of bruises or disease were chosen as experimental material. The in vivo antifungal efficacy of HFF extracts to control citrus blue mold (*P. italicum*) was carried out according to our previous study [21]. All selected fruits were dipped in 1.0% (*v*/*v*) sodium hypochlorite solution for 1 min, washed with running tap H_2_O to remove the residual disinfectant, and air-dried on a sterilized bench top before wounding. The wounds were injected with 15 µL of PEE, CE, EAE, AE, ME, WE, and sterile water (as control). After 60 min at room temperature, each wound was reinjected with 15 µL of *P. italicum* suspension adjusted to 10^6^ cfu/mL. There were three replicate trials of twelve fruits per treatment, and all experiments were performed twice, obtaining consistent data. The treated and control fruits were placed in a plastic crate with a sealed plastic bag and incubated at 27 °C and ~95% relative humidity (RH) for 7 days. Blue mold inhibition at the unified concentration (10 mg/mL) of different HFF extracts was formulated using the below equation.

$$\mathrm{Blue}\;\mathrm{mold}\;\mathrm{inhibition}\;(\%)\;=\;\frac{dc-dt}{dc}\times 100$$

where d*c* and d*t* were the averages of lesion diameters (mm) in the control and HFF extracts treatment, respectively.

### 2.7. HPLC-QTOF-MS Analysis

High performance liquid chromatography coupled with quadrupole time-of-flight mass spectrometry (HPLC-QTOF-MS) analysis was performed using an Agilent 1290–6540 liquid chromatography system connected to a time-of-flight mass spectrometer (Agilent technologies, CA, USA). Separations were performed on an Agilent Extend C18 (2.1 × 50 mm, 1.8 µm) column. The mobile phase consisted of A (0.1% formic acid in water) and B (0.1% formic acid in acetonitrile). A linear gradient program at a flow rate of 0.40 mL/min was used: 0 to 2 min, from 10% to 10% (B); 2 to 6 min, 10% to 90% (B); 6 to 9 min, 90% to 90% (B); 9 to 10 min, 90% to 10% (B), and then followed by 10% (B) for 2 min. MS spectra were acquired by full range acquisition covering *m/z* 100–1000. Metabolites were identified according to standard samples or by comparison with data from the literature. The Agilent MassHunter Qualitative B.07 Software was used for instrument control, data acquisition, and data analysis. Each sample was investigated in both negative and positive ion mode with the following parameters. Negative source parameters were as follows: spray voltage 4 kV with a desolvation temperature of 350 °C, drying gas 11 L/min, and nebulizer 310 kPa. MS spectra were acquired by full range acquisition covering *m/z* 100–1000. Positive source parameters were as follows: spray voltage 3.5 kV with a desolvation temperature of 350 °C, drying gas 11 L/min, and nebulizer 310 kPa. MS spectra were acquired by full-range acquisition covering *m/z* 100–1000.

### 2.8. Statistical Analysis

The statistical analyses were done by using the SPSS 17.0 software package (SPSS Inc., Chicago, IL, USA). The data obtained is presented as the means ± standard deviation (SD) of three independent readings. Duncan’s multiple range tests were applied to determine the mean differences. The differences at *p* < 0.05 were set as statistically significant.

## 3. Results and Discussion

### 3.1. Extract Yields, TPC, and TFC of HFF Extracts

The yields of various solvent extracts from HFF obtained with increasing polarity were ranged from 1.29% ± 0.09% (petroleum ether extract, PEE) to 8.34% ± 0.32% (water extract, WE) (Table 1). Among the six different HFF extracts, the WE showed the highest yield, possibly due to its fascinating amounts of water-soluble components (polysaccharides, organic acids, proteins, etc.). Numerous reports have clearly shown a close association among the polarity of extraction solvent and the extract yields; the higher the polarity, the higher the extract yields [22,23]. Herein, the yield values of HFF extracts showed that the WE had the highest yield (8.34% ± 0.32%) followed by ME (3.59% ± 0.29%), AE (2.79% ± 0.21%), CE (1.77% ± 0.12%), EAE (1.71% ± 0.08%), and PEE (1.29% ± 0.09%) (Table 1). There exists a positive but significant correlation with the polarity of extract solvent (*R*^2^ = 0.899, *p* < 0.05). Lim and co-workers [24] showed that yields of various solvent extracts from *Sargassum serratifoliumis* increased with increasing the relative polarity of the extraction solvent, and the current results showed a mimicking trend, where WE showed the highest yield compared to the other five extracts. Medium polarity solvents, ethyl acetate and acetone, were suitable for the extraction of TFC and TPC from HFF. The high polarity solvents, methanol and water, were suitable for the extraction of water-soluble components, and the nonpolar solvents, petroleum ether and chloroform, were suitable for the extraction of essential oils, terpenoids, lipids, and chlorophyll.

The TPC and TFC of all HFF extracts were measured by a spectrophotometric method, and the results are shown in Table 1. The considerable differences of TPC were found in different solvent extracts, of which AE showed the highest TPC (85.25 ± 1.72 mg GAE/g dw), and this level was 2.56, 2.78, 2.95, and 4.80 times higher than that in CE (33.36 ± 1.16 mg GAE/g dw), ME (30.68 ± 0.66 mg GAE/g dw), WE (28.87 ± 0.88 mg GAE/g dw), and PEE (17.75 ± 0.52 mg GAE/g dw), respectively. The TFC level ranged from 15.80 to 144.22 mg RE/g dw. The highest TFC was detected in AE (144.22 ± 8.46 mg RE/g dw), followed by CE (102.56 ± 6.48 mg RE/g dw), EAE (81.74 ± 4.04 mg RE/g dw), PEE (26.69 ± 1.93 mg RE/g dw), ME (24.07 ± 1.28 mg RE/g dw), and WE (15.80 ± 0.59 mg RE/g dw). The WE exhibited the highest extract yield (8.34% ± 0.32%) but the lowest TFC. Interestingly, the TPC and TFC in HFF extracts of petroleum ether, chloroform, ethyl acetate, and acetone showed a significantly positive correlation with the polarity of extract solvents (*R*^2^ = 0.94 and 0.88, respectively).

Moreover, the AE of HFF in our present study had the highest TFC and showed a good positive correlation with the IC_50_ (2.02 mg/mL) of DPPH radical-scavenging activity (Table 2). The result was consistent with the previously reported fact that the isopropanol extract from *F. hirta* Vahl. roots has 316.65 mg/g of TPC with an IC_50_ (4.53 mg/mL) of DPPH radical-scavenging activity [25].

### 3.2. In Vitro Antioxidant Activities of HFF Extracts

**Figure 1:**
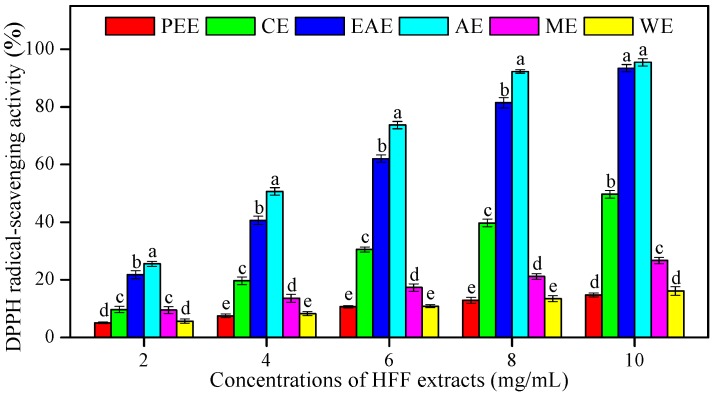
1,1-diphenyl-2-picrylhydrazyl (DPPH) radical-scavenging activity of HFF extracts with various concentrations (2, 4, 6, 8, and 10 mg/mL). Mean ± standard deviation (SD, *n* = 3) with the different lowercases (a, b, c, d, and e, respectively) were significantly different (*p* < 0.05) using Duncan’s test. PEE: petroleum ether extract; CE: chloroform extract; EAE: ethyl acetate extract; AE: acetone extract; ME: methanol extract; WE: water extract.

The in vitro antioxidant activities of HFF extracts were estimated using the DPPH, ABTS, and FRAP assays, which increased in a concentration-dependent manner. A significant dose-dependent inhibition in DPPH radical scavenging was observed for HFF extracts. The higher concentration of HFF extracts, the higher the percentage inhibition (Figure 1). The IC_50_ values of radical-scavenging activity of HFF extracts (Table 2) was observed and found in the following order: AE (2.02 mg/mL) < EAE (2.52 mg/mL) < CE (5.06 mg/mL) < ME (9.58 mg/mL) < WE (16.90 mg/mL) < PEE (19.06 mg/mL). The IC_50_ value of AsA was used as an antioxidant standard (0.13 mg/mL). A high correlation of IC_50_ was observed with TPC (*R*^2^ = 0.82, *p* < 0.05) as well as TFC (*R*^2^ = 0.84, *p* < 0.05) (Table 3).

**Figure 2:**
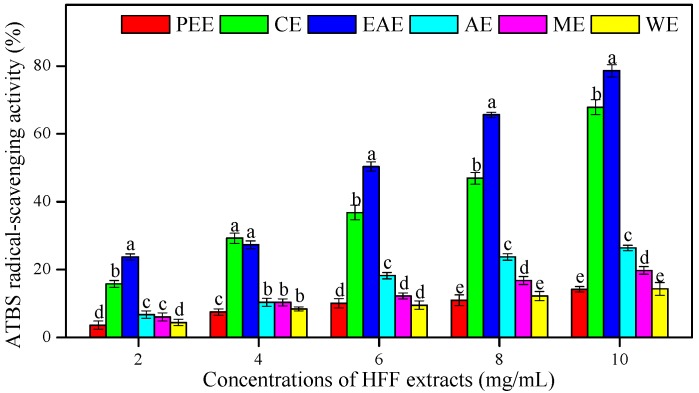
The ATBS (2,2-azino-bis-(3-ethylbenzthiazoline-6-sulphonic acid) radical-scavenging activity of HFF extracts with various concentrations (2, 4, 6, 8, and 10 mg/mL). Mean ± standard deviation (SD, *n* = 3) with the different lowercases (a, b, c, d, and e, respectively) were significantly different (*p* < 0.05) using Duncan’s test.

The ATBS (2,2-azino-bis-(3-ethylbenzthiazoline-6-sulphonic acid) radical-scavenging effects of HFF extracts were measured in different concentrations (2–10 mg/mL) and compared to AsA (Figure 2 and Table 4). The EAE showed the highest ATBS radical-scavenging activity and the lowest IC_50_ (3.06 mg/mL), followed by CE (IC_50_ = 3.87 mg/mL), AE (IC_50_ = 9.26 mg/mL), ME (IC_50_ = 13.92 mg/mL), WE (IC_50_ = 20.12 mg/mL), and PEE (IC_50_ = 23.69 mg/mL), as compared to AsA used as an antioxidant standard (0.16 mg/mL). The IC_50_ values of HFF extracts showed a positive and strong correlation with TFC (*R*^2^ = 0.83, *p* < 0.05) (Table 3).

**Figure 3:**
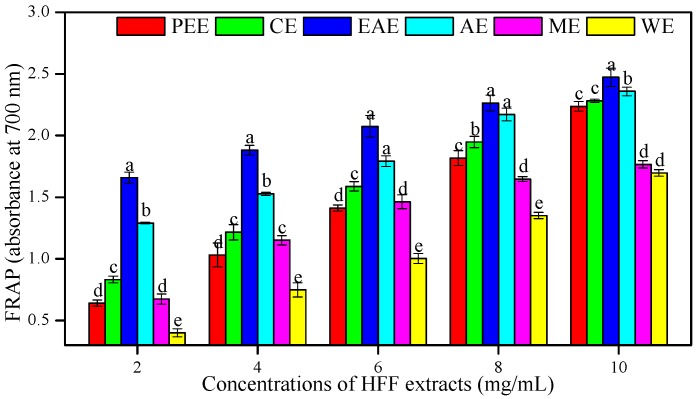
Ferric reducing antioxidant potential (FRAP) of HFF extracts with various concentrations (2, 4, 6, 8, and 10 mg/mL). Mean ± standard deviation (SD, *n* = 3) with the different lowercases (a, b, c, d, and e, respectively) were significantly different (*p* < 0.05) using Duncan’s test.

For the FRAP assay, the absorbance at 700 nm of six HFF extracts was increased with their concentrations and indicated a higher antioxidant potential. The EAE showed the best antioxidant potential to reduce ferric ions than other HFF extracts (*p* < 0.05), followed by AE, CE, PEE, ME, and WE (Figure 3). The FRAP of HFF extracts showed a highly significant correlation with TPC (*R*^2^ = 0. 92, *p* < 0.01) (Table 3).

It is generally proven that the antioxidant activity of plants depends on the amounts of biologically active compounds (flavonoids, phenolics, phenylpropanoids, etc.). Generally, the higher the TPC and TFC, the stronger antioxidant and antifungal activities reported [16,18,26]. In the current study, the various solvent extracts of HFF were taken for analysis, where AE showed the higher antioxidant activity in DPPH radical-scavenging assay, and EAE exhibited the stronger antioxidant activities in ABTS radical-scavenging and the FRAP assay. Current findings indicated that a significant correlation of antioxidant activity with TPC and TFC in HFF extracts occurred, which helps us explain that HFF has abundant promising natural antioxidants that retard cell aging and inflammations [2,3,14]. As shown in Table 5, five flavonoid constituents, namely pinocembrin-7-O-β-D-glucoside, naringenin-7-O-β-D-glucoside, eriodictyol-7-O-β-D-glucoside, luteolin, and apigenin, were all detected in EAE, AE, and ME. Those flavonoids were highly contributing to antioxidant activity. Unfortunately, all HFF extracts (IC_50_ values of the DPPH and ATBS radical-scavenging assay) showed significantly weaker antioxidant activity compared to the standard of ascorbic acid with the IC_50_ values of 0.13 and 0.16 mg/mL, respectively (Table 2 and Table 4). A recent study [27] showed that the FRAP and DPPH radical-scavenging activity of the ethanol extract of *Melissa officinalis* L. leaves were lower than ascorbic acid (control), which were consistent with our present findings. However, the current study has confirmed that the in vitro antioxidant activities of HFF are stronger than *Impatiens balsamina* L. [18], *Agastache rugosa* (Korean mint) [28], and *Incarvillea compacta* Maxim [29].

### 3.3. In Vitro Antifungal Activities of HFF Extracts

The antifungal activity of HFF extracts were examined against six different fungal strains (*P. italicum*, *P. digitatum*, *A. niger*, *A. oryzae*, *S. cerevisiae*, and *C. utilis*), and their antifungal potencies were examined qualitatively by the presence or absence, and the diameters, of inhibition zones. The results obtained for the in vitro antifungal activity of HFF extracts against the above six pathogenic fungi are presented in Table 6. Extraction solvents alone did not have antifungal activity. The results showed that HFF extracts (10 mg/mL) had varying degrees of in vitro antifungal activity (diameters ranged from 12.25 ± 0.96 mm to 41.75 ± 0.96 mm, while natamycin (0.05 mg/mL) as the positive control was 22.75 ± 1.50 mm to 27.20 ± 1.50 mm) against the tested fungal strains. The AE of HFF had an excellent activity against *P. italicum*, *P. digitatum*, *A. oryzae*, and *S. cerevisiae*, and fairly strong activity against *A. niger* and *C. utilis*, whereas the EAE had a strong activity against *A. niger* and *C. utilis*. The WE of HFF showed a weaker in vitro antifungal activity against two *Penicillium* strains (*P. italicum* and *P. digitatum*). However, it did not possess any in vitro antifungal activity against two *Aspergillus* strains (*A. niger* and *A. oryzae*), *S. cerevisiae*, and *C. utilis* (Table 6). There was in vitro antifungal activity of HFF extracts against *P. italicum*, *P. digitatum*, *A. oryzae*, and *S. cerevisiae*, which exhibited a highly significant correlation with TFC (*R*^2^ = 0.87, 0.86, 0.88, and 0.82, *p* < 0.05) (Table 3).

The six tested fungal strains (*P. italicum*, *P. digitatum*, *A. niger*, *A. oryzae*, *S. cerevisiae*, and *C. utilis*) were inhibited at a concentration of 2000 µg/mL of CE, EAE, and AE of HFF, whereas AE was a more effective inhibitor at a concentration of 1000 µg/mL (Table 7). The two *Penicillium* strains (*P. italicum* CGMCC 3.4040 and *P. digitatum* CGMCC 3.15410) and two *Aspergillus* strains (*A. niger* CGMCC 3.17612 and *A. oryzae* CGMCC 3.13905) were found to be more susceptible to the AE with lower MIC values of 125 µg/mL, 250 µg/mL, 500 µg/mL, and 250 µg/mL, respectively (Table 7). Based on this result, it could be concluded that the main antifungal components which contributed to the antifungal activity of HFF are enriched in AE of HFF. As shown in Table 5, the antifungal compound pinocembrin-7-*O*-β-D-glucoside [11] was detected in AE and EAE.

As it is well known, *Penicillium* spp., *Aspergillus* spp., *S. cerevisiae*, and *C. utilis* have been identified as the causal agents of plant-borne diseases [11,30]. The potential of application for plant extracts is the inhibition of fungal growth and reduction in the incidence of diseases in horticultural crops [10,11,21,31]. As far as we know, most of the previous scientific studies have been focused mainly on the roots, neglecting the fruits of *F. hirta* Vahl. Therefore, the fruits may be considered to be a natural preservative against plant-borne pathogens for the horticultural production industry.

### 3.4. In Vivo Antifungal Activity of HFF Extracts on Blue Mold Development on Inoculated Fruit

**Figure 4:**
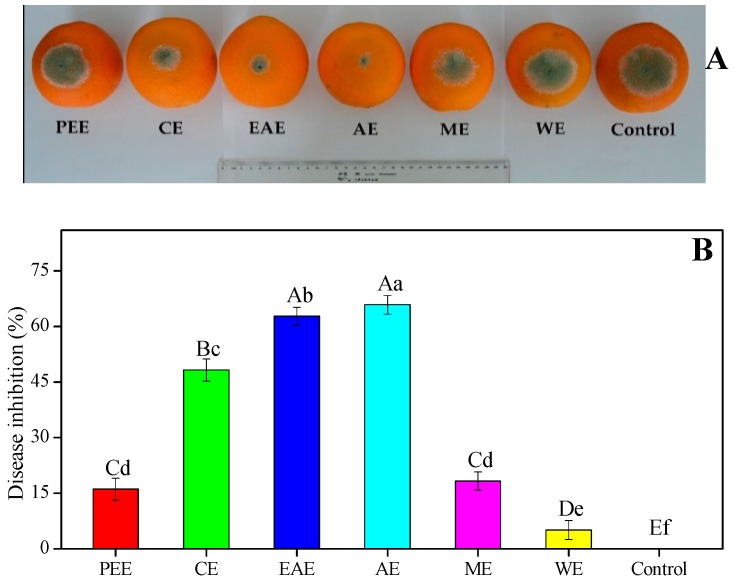
Antifungal efficacy of HFF extracts on in vivo mycelial growth of *Penicillium italicum* in ‘Lane Late’ navel oranges. (**A**) Blue mold development and (**B**) disease inhibition were measured after the incubation of 7 days at 27 °C. Bars indicate that the mean ± standard deviation (SD, *n* = 12) with different upper and lower case letters (A–E and a–f) were significantly different (*p* < 0.01 and *p* < 0.05, respectively) according to Duncan’s multiple range test, respectively.

The in vivo antifungal efficacy of different HFF extracts in reducing the disease development caused by blue mold on artificially inoculated ‘Lane Late’ navel oranges was well elucidated (Figure 4). The disease development and lesion diameter of citrus blue mold were significantly more reduced by treatment of most of HFF extracts than the control group after following the incubation for 7 days at 27 °C (Figure 4). The AE of HFF at 10 mg/mL exhibited the lowest lesion diameter (19.38 ± 1.41 mm) and the highest disease inhibition (65.86% ± 2.48%) compared with the control (Figure 4A, B). The CE, ME, and PEE of HFF had a moderate in vivo antifungal efficacy on blue mold, the lesion diameter of *P. italicum* rot varying between 29.38 ± 1.68 mm and 47.63 ± 1.41 mm, and the mold growth inhibition (MGI) ranging between 16.08% ± 2.39% and 48.28% ± 2.97%. Based on the present study, HFF extracts possessed potential antifungal activities, particularly the in vivo antifungal activity against *P. italicum*, which is highly in agreement with a published study by Su et al. [18] who reported that *Impatiens balsamina* L. stem extracts obtained with various solvents possessed significant antimicrobial activity against six fungal strains and four bacterial strains. This was highly in line with our previous study, which revealed the in vivo antifungal efficacy of the ethanol extract from HFF for controlling *P. italicum* rot in citrus fruit, such as Nanfeng mandarins and Xinyu tangerines [7,21].

Recently, the screening and isolation of botanical fungicides against citrus postharvest fungal diseases and the inhibitory effects have gained much attention [31,32,33,34,35,36]. HFF was considered to be a very promising source of health promotion being used and witnessed by the Hakka people in southern China as a folk medicine for the treatment of invigorating spleen, supplementing Qi, eliminating dampness, promoting lactation, and lessen the inflammations [2,5,14,15]. Only a few studies have focused on the antifungal activity of HFF against plant fungi. The in vitro trials of plant extracts is an essential and initial step for screening botanical fungicides with potential activity against plant pathogenic fungi, hence in vivo trials are needed to check whether the positive results of the in vitro trials can be useful for future studies [37,38,39]. The in vitro antifungal activity using six different HFFE and their in vivo efficacy to control postharvest blue mold of citrus fruits were examined. Among the six HFF extracts, AE showed the highest (41.75 ± 0.96 mm and 65.86% ± 2.48%) antifungal activity in both in vitro and in vivo trials, followed by that of EAE (41.00 ± 0.82 mm and 62.78% ± 2.37%) (Table 6 and Figure 4). Interestingly, in vitro antifungal activity of ethanol extract of HFF against plant fungal growth was tested previously [10], but the highest antifungal activity of AE could be attributed to the high levels of TFC that are widely reported to achieve a remarkable effect of antimicrobial activity [18,22,28]. In line with current experimental data, a recent study was reported by Chen and Ye [9], who claimed that aqueous extracts of *F. hirta* Vahl. roots against *Escherichia coli* and *Staphylococcus aureus* have the best antibacterial activity. Moreover, the in vivo trials confirmed the strong antifungal activity shown in vitro by the AE of HFF. It proved to be highly effective in controlling postharvest blue mold incidence of Nanfeng mandarins and Xinyu tangerines infected by *P. italicum* [7,21].

### 3.5. HPLC-QTOF-MS Analysis of HFF Extracts

A total of eighteen secondary metabolites were identified or putatively identified from six HFF extracts (Table 5). Fifteen metabolites were identified by comparison of the retention time (RT) and extracted positive or negative ions with standard samples previously isolated and identified by NMR from extracts of *F. hirta* Vahl. [8,11]. Three phenolics, namely, luteolin, apigenin, and umbelliferone, were putatively identified by comparison of extracted positive or negative ions with literature data, for which those were the main constituents previously reported [2,3]. Five flavonoid constituents (13–17) were all detected in EAE, AE, and ME of HFF. Furthermore, pinocembrin-7-*O*-β-D-glucoside (13), the main antifungal flavonoid, was also detected in CE of HFF. Two carboline alkaloids (1 and 2) were all detected in polar extracts (AE, ME, WE), while sesquiterpenoids/norsesquiterpenoids (3–6) and monosubstituted benzene derivatives (7–12) were detected in most HFF extracts, except PEE. Only three compounds were detected in PEE extract, notably, umbelliferone (18) was only detected in CE extract.

## 4. Conclusions

The present study demonstrated that various extraction solvents (petroleum ether, chloroform, ethyl acetate, acetone, methanol, and distilled water) used for HFF, affected extract yields, total phenolic and flavonoid contents, antioxidant and antifungal activities, as well as the chemical constituents. The CE and AE of HFF showed relatively higher levels of TPC, TFC, and antioxidant activities, which explain why they had a high antifungal potential against *P. italicum*, *P. digitatum*, *A. oryzae*, and *S. cerevisiae* using agar diffusion. Greater efficacy on reducing blue mold in citrus fruits has been observed when compared with other extracts during in vivo trials. Eighteen secondary metabolites including flavonoids, carboline alkaloids, monosubstituted benzene derivatives, and sesquiterpenoids were identified or putatively identified from six HFF extracts. However, further in-depth studies of chemical constituents are needed to isolate and confirm antifungal compounds in various HFF extracts that are responsible for the active mechanism. It is of common interest and with great potential for natural antifungal compounds isolated from plants to be used as novel alternatives against synthetic fungicides for helping to control postharvest fungal diseases of citrus fruit or related agricultural crops.

## Figures and Tables

**Table 1:** Extract yields, total phenolic contents (TPC) and total flavonoid contents (TFC) of hairy fig fruits (HFF) obtained by various solvents.

Extract Solvents	Polarity	Yield (%)	TPC (mg GAE/g dw)	TFC (mg RE/g dw)
Petroleum ether	0.01	1.29 ± 0.09 ^e^	17.75 ± 0.52 ^e^	26.69 ± 1.93 ^d^
Chloroform	2.60	1.77 ± 0.12 ^d^	33.36 ± 1.16 ^b^	102.56 ± 6.48 ^b^
Ethyl acetate	4.30	1.71 ± 0.08 ^d^	84.03 ± 2.29 ^a^	81.74 ± 4.04 ^c^
Acetone	5.40	2.79 ± 0.21 ^c^	85.25 ± 1.72 ^a^	144.22 ± 8.46 ^a^
Methanol	6.60	3.59 ± 0.29 ^b^	30.68 ± 0.66 ^c^	24.07 ± 1.28 ^d^
Water	10.20	8.34 ± 0.32 ^a^	28.87 ± 0.88 ^d^	15.80 ± 0.59 ^e^

GAE: gallic acid equivalent; RE: rutin equivalent; dw: dry weight. Mean ± standard deviation (SD, *n* = 3) with the different lowercases (a, b, c, d, and e, respectively) were significantly different (*p* < 0.05) using Duncan’s test in each column.

**Table 2:** The regression equations, correlation coefficient (*R*^2^), and IC_50_ values of the DPPH radical-scavenging activities of six HFF extracts (concentrations ranged from 2 to 10 mg/mL) and ascorbic acid (concentrations ranged from 0.02 to 0.2 mg/mL).

Extract Solvents	Regression Equations	*R* ^2^	IC_50_ (mg/mL)
Petroleum ether	y = 2.4799x + 2.7274	0.9922	19.06
Chloroform	y = 9.6033x + 1.4601	0.9983	5.06
Ethyl acetate	y = 17.019x + 7.1932	0.9792	2.52
Acetone	y = 22.323x + 4.8447	0.9917	2.02
Methanol	y = 4.6913x + 5.0789	0.9878	9.58
Water	y = 2.8148x + 2.4214	0.9792	16.90
Ascorbic acid	y = 378.49x + 0.2104	0.9991	0.13

**Table 3:** Correlations of antioxidants and antifungals against six strains with TPC and TFC, respectively.

	Index	Correlation *R*^2^	
		TPC	TFC
Antioxidants	DPPH scavenging activity	0.82 *	0.84 *
	ABTS scavenging activity	0.67	0.83 *
	FRAP assay	0.92 **	0.70
Antifungals	*P. italicum*	0.74	0.87 *
	*P. digitatum*	0.80	0.86 *
	*A. niger*	0.78	0.78
	*A. oryzae*	0.72	0.88 *
	*S. cerevisiae*	0.68	0.82 *
	*C. utilis*	0.84 *	0.77

Each column with * and ** was significantly different at *p* < 0.05 and *p* < 0.01, respectively.

**Table 4:** The regression equations, correlation coefficient (*R*^2^), and IC_50_ values of the ATBS radical-scavenging activities of six HFF extracts (concentrations ranged from 2 to 10 mg/mL) and ascorbic acid (concentrations ranged from 0.05 to 0.2 mg/mL).

Extract Solvents	Regression Equations	*R* ^2^	IC_50_ (mg/mL)
Petroleum ether	y = 1.9809x + 3.1291	0.9801	23.69
Chloroform	y = 12.189x + 2.7832	0.9674	3.87
Ethyl acetate	y = 14.121x + 6.7744	0.9655	3.06
Acetone	y = 5.2587x + 1.3240	0.9760	9.26
Methanol	y = 3.3853x + 2.8794	0.9885	13.92
Water	y = 2.3497x + 2.7319	0.9732	20.12
Ascorbic acid	y = 296.09x + 1.6043	0.9993	0.16

**Table 5:** Secondary metabolites of HFF Extracts analysis by HPLC-QTOF-MS.

No.	Name	Molecular Formula	Exact Mass	RT (min)	M + H	M − H	Extracts					
							PEE	CE	EAE	AE	ME	WE
1	methyl-1,2,3,4-tetrahydro-*β*-carboline-3-carboxylic acid	C_13_H_14_N_2_O_2_	230.1055	0.73	231.1128	229.0975				+	+	+
2	methyl-1-methyl-1,2,3,4-tetrahydro-*β*-carboline-3-carboxylate	C_14_H_16_N_2_O_2_	244.1212	1.60	245.1285				+	+	+	+
3	dihydrophaseic acid	C_15_H_22_O_5_	282.1467	0.82		281.1388	+	+	+	+	+	+
4	vomifoliol	C_13_H_20_O_3_	224.1412	1.17	225.1505			+	+	+	+	+
5	dehydrovomifoliol	C_13_H_18_O_3_	222.1256	1.77	223.1329			+	+	+	+	+
6	pubinernoid A	C_11_H_16_O_3_	196.1099	1.47	197.1177		+	+	+	+	+	+
7	2-phenylethyl-*O*-*β* d-glucoside	C_14_H_20_O_6_	284.1260	1.15	285.1329			+		+	+	+
8	1-*O*-trans-cinnamoyl-*β*-D-glucopyranosyl-(1→6)-*β*-d-glucopyranoside	C_21_H_28_O_12_	472.1581	4.56	473.1656		+	+	+	+	+	+
9	4-*O*-benzoyl-quinic acid	C_14_H_16_O_7_	296.0896	0.99	297.0970	295.0817		+	+	+	+	+
10	3-*O-*benzoyl-quinic acid	C_14_H_16_O_7_	296.0896	0.73	297.0973	295.0818		+	+	+	+	+
11	benzyl-*β*-d-glucopyranoside	C_13_H_18_O_6_	270.1103	0.60		269.1027				+	+	+
12	(2S) 2-*O*-benzoyl-butanedioic acid-4-methyl ester	C_12_H_12_O_6_	252.0634	3.77	253.0712			+				
13	pinocembrin-7-*O*-*β*-d-glucoside	C_21_H_22_O_9_	418.1264	3.59		417.1189		+	+	+	+	
14	naringenin-7-*O*-*β*-d-glucoside	C_21_H_22_O_10_	434.1213	2.09		433.1126			+	+	+	
15	eriodictyol-7-*O*-*β*-d-glucoside	C_21_H_22_O_11_	450.1162	1.23		449.1084			+	+	+	
16	luteolin	C_15_H_10_O_6_	286.0477	3.92		285.0399			+	+	+	
17	apigenin	C_15_H_10_O_5_	270.0528	3.94	271.0614				+	+	+	
18	umbelliferone	C_9_H_6_O_3_	162.0317	3.72	163.0389			+				

+ Secondary metabolites were presented in extracts.

**Table 6:** Inhibition effects of HFF extracts at 10 mg/mL and natamycin against six fungi.

Fungi	Inhibition Zone (mm)						
	PEE	CE	EAE	AE	ME	WE	Natamycin
*P. italicum*	27.50 ± 0.58 ^c^	37.50 ± 1.29 ^b^	41.00 ± 0.82 ^a^	41.75 ± 0.96 ^a^	28.00 ± 0.80 ^c^	15.50 ± 1.00 ^d^	27.20 ± 1.50 ^c^
*P. digitatum*	20.00 ± 0.82 ^d^	24.25 ± 1.89 ^c^	28.00 ± 2.16 ^b^	31.50 ± 1.29 ^a^	21.50 ± 1.29 ^d^	12.25 ± 0.96 ^e^	24.70 ± 0.96 ^c^
*A. niger*	17.75 ± 0.96 ^e^	25.00 ± 1.41 ^c^	37.25 ± 1.26 ^a^	33.25 ± 1.26 ^b^	17.50 ± 0.58 ^e^	—	27.00 ± 0.82 ^d^
*A. oryzae*	14.50 ± 1.29 ^d^	20.00 ± 0.82 ^c^	24.00 ± 1.15 ^b^	28.00 ± 1.41 ^a^	—	—	24.20 ± 0.50 ^b^
*S. cerevisiae*	19.75 ± 0.50 ^d^	23.50 ± 1.00 ^c^	27.75 ± 0.96 ^b^	31.50 ± 1.29 ^a^	14.25 ± 0.96 ^e^	—	24.50 ± 1.00 ^c^
*C. utilis*	—	14.50 ± 1.29 ^c^	22.00 ± 0.82 ^a^	19.50 ± 1.00 ^b^	12.50 ± 1.00 ^d^	—	22.75 ± 1.50 ^a^

Mean ± standard deviation (SD, *n* = 4) with the different lowercases (a, b, c, d, and e, respectively) were significantly different (*p* < 0.05) using Duncan’s test in each row; natamycin at 0.05 mg/mL as positive control. —: No inhibition zone.

**Table 7:** Minimal inhibitory concentration (MIC) of various solvent extracts from HFF and natamycin against six fungi.

Fungi Strains	MIC ^a^						
	PEE	CE	EAE	AE	ME	WE	Natamycin ^b^
*P. italicum*	1000	250	125	125	500	2000	2
*P. digitatum*	1000	500	500	250	1000	NT	2
*A. niger*	2000	1000	500	500	2000	NT	2
*A. oryzae*	1000	500	500	250	NT	NT	2
*S. cerevisiae*	1000	1000	1000	500	2000	NT	4
*C. utilis*	NT	2000	1000	1000	2000	NT	4

NT: not tested. ^a^: minimal inhibition concentrations (μg/mL); ^b^: natamycin as positive control.
